# Analysis of serious adverse events in a paediatric fast breathing pneumonia clinical trial in Malawi

**DOI:** 10.1136/bmjresp-2019-000415

**Published:** 2019-09-03

**Authors:** Evangelyn Nkwopara, Robert Schmicker, Tisungane Mvalo, Melda Phiri, Ajib Phiri, Mari Couasnon, Eric D. McCollum, Amy Sarah Ginsburg

**Affiliations:** 1 International Programs, Save the Children Federation Inc, Fairfield, Connecticut, USA; 2 Department of Biostatistics, University of Washington, Seattle, Washington, USA; 3 Department of Pediatrics, University of North Carolina Project, Lilongwe Medical Relief Fund Trust, Lilongwe, Malawi; 4 Department of Pediatrics and Child Health, University of Malawi College of Medicine, Blantyre, Malawi; 5 Eudowood Division of Pediatric Respiratory Sciences, Department of Pediatrics, Johns Hopkins School of Medicine, Baltimore, Maryland, USA

**Keywords:** pneumonia

## Abstract

**Introduction:**

Pneumonia is the leading infectious killer of children. We conducted a double-blind, randomised controlled non-inferiority trial comparing placebo to amoxicillin treatment for fast breathing pneumonia in HIV-negative children aged 2–59 months in Malawi. Occurrence of serious adverse events (SAEs) during the trial were examined to assess disease progression, co-morbidities, recurrence of pneumonia and side effects of amoxicillin.

**Methods:**

Enrolled children with fast breathing for age and a history of cough <14 days or difficult breathing were randomised to either placebo or amoxicillin for 3 days, and followed for 14 days to track clinical characteristics and outcomes. Medical history, physical exam, laboratory results and any chest radiographs collected at screening, enrolment and during hospitalisation were evaluated. All SAE reports were reviewed for additional information regarding hospitalisation, course of treatment and outcome.

**Results:**

In total, 102/1126 (9.0%) enrolled children with fast breathing pneumonia were reported to have a SAE. Seventy-five per cent (n=77) of SAEs were pneumonia-related (p<0.01). Children<2 years of age represented the greatest proportion (61/77, 79.2%) of those with a pneumonia-related SAE. In the amoxicillin group, there were 46 SAEs and 5 (10.9%) cases were identified as possibly related to study drug (4 gastroenteritis and 1 fever). There were no life-threatening pneumonia SAEs or deaths in either group, and by the time of exit from the study, all children recovered without sequelae.

**Discussion:**

In this fast breathing pneumonia clinical trial, SAEs occurred infrequently in both the amoxicillin and placebo groups, and amoxicillin was well tolerated.

**Trial registration number:**

NCT02760420. https://clinicaltrials.gov/ct2/show/NCT02760420?term=ginsburg&rank=9.

**Key messages**

- What are the safety implications when conducting a paediatric fast breathing pneumonia clinical trial involving placebo in a malaria-endemic setting in Africa?
- By carefully adhering to strict eligibility criteria and conducting frequent and careful clinical monitoring of enrolled children with fast breathing pneumonia, our study population in Lilongwe, Malawi experienced no deaths, no life-threatening events and an overall low incidence of serious adverse events.
- This secondary analysis of safety outcomes demonstrates that it is possible to safely conduct a large-scale paediatric pneumonia clinical trial in which placebo is involved.

## Introduction

Globally, pneumonia remains the leading infectious cause of death in children aged less than 5 years.^1^ Although the number of child deaths due to pneumonia has dropped significantly in the last 15 years, more work is needed to improve the diagnosis and treatment of pneumonia, particularly in sub-Saharan Africa.^2^ WHO Integrated Management of Childhood Illness (IMCI) guidelines were developed to be pragmatic and to assist with identifying and treating acutely ill children in low resource settings using mainly clinical signs rather than invasive testing or imaging.^3^ Current WHO IMCI guidelines diagnose pneumonia by identifying fast breathing and/or chest indrawing in a child with cough or difficult breathing, and recommend outpatient treatment with amoxicillin for those without accompanying clinical danger signs. However, it has been questioned whether fast breathing as an isolated clinical sign in a child with cough or difficult breathing is due to bacteria and requires antibiotics.^4^ We recently completed a double-blind randomised controlled non-inferiority trial in HIV-uninfected children aged 2–59 months with WHO IMCI-defined fast breathing pneumonia in a malaria-endemic region of Malawi which showed treatment with placebo was inferior to 3 days of treatment with amoxicillin.^5^ Given the opportunity to assess clinical outcomes in both a placebo group and a group receiving antibiotic treatment, we examined the incidence of serious adverse events (SAEs) within each group as a secondary analysis.

While the safety of amoxicillin use in children with pneumonia has been established, amoxicillin has important side effects including diarrhoea, nausea, vomiting, fever and rash as well as rarer but more serious side effects such as abnormal liver function tests, interstitial nephritis, seizures and Stevens-Johnson syndrome, all of which could lead to SAEs.^6 7^ In the absence of antibiotic treatment, there is concern for clinical deterioration and SAEs in children with bacterial infections like pneumonia. While this secondary analysis is not a reassessment of amoxicillin’s safety or effectiveness, there is value in assessing both the harms and benefits of amoxicillin, particularly in a randomised controlled trial that includes a placebo group. We also investigate the impact, if any, on disease progression in the placebo group since these children did not receive antibiotics for WHO-defined pneumonia despite having clinical signs.

## Methods

HIV-uninfected children aged 2–59 months who presented with fast breathing for age in the presence of a cough <14 days or difficult breathing were enrolled (table 1). The study was conducted at Kamuzu Central Hospital (KCH) and Bwaila District Hospital in Lilongwe, Malawi. Final eligibility determination for enrolment depended on the results of the medical history, physical examination, laboratory testing, appropriate understanding of the study and completion of the written informed consent process. Enrolled children were randomised to either 3 days of amoxicillin or placebo dispersible tablet treatment, and then followed for 14 days to track clinical outcomes. At follow-up visits, children were assessed for treatment failure (primary endpoint, day 4) or clinical relapse.

**Table 1:** Study terms and definitions

Fast breathing for age	Respiratory rate >50 breaths per minute for children t2 to <12 months of age, or >40 breaths per minute for children>12 months of age
Severe respiratory distress	Grunting, nasal flaring, head nodding and/or chest indrawing
Hypoxaemia	Arterial oxyhaemoglobin saturation (SpO_2_) <90% in room air, as assessed non-invasively by a pulse oximeter
WHO IMCI general danger signs	Lethargy or unconsciousness, convulsions, vomiting everything or inability to drink or breastfeed
Severe acute malnutrition	Weight for height/length <-3 SD, MUAC <11.5 cm, or peripheral oedema
Severe malaria	Positive malaria rapid diagnostic test with any WHO IMCI general danger sign, stiff neck, abnormal bleeding, clinical jaundice, or haemoglobinuria
HIV exposure	Children<24 months of age with a HIV-infected mother
Serious adverse event	Adverse event that: Results in death Is life threatening Requires inpatient hospitalisation or prolongation of existing hospitalisation Results in persistent or significant disability/incapacity Is a medical event, based on appropriate medical judgement, that may jeopardise the health of the participating child or require medical or surgical intervention to prevent one of the outcomes listed
**Eligibility criteria**	
Inclusion criteria	2–59 months of age Cough <14 days or difficulty breathing Fast breathing for age
Exclusion criteria	Severe respiratory distress Hypoxaemia Resolution of fast-breathing after bronchodilator challenge, if wheezing at screening examination WHO IMCI general danger signs Stridor when calm HIV seropositivity or HIV exposure Severe acute malnutrition Possible tuberculosis (coughing >14 days) Anaemia with haemoglobin <80 g/L Severe malaria Known allergy to penicillin or amoxicillin Receipt of an antibiotic treatment in the 48 hours prior to the study Hospitalised within 14 days prior to the study Living outside the study area Any medical or psychosocial condition or circumstance that, in the opinion of the investigators, would interfere with the conduct of the study or for which study participation might jeopardise the child’s health Any non-pneumonia acute medical illness which requires antibiotic treatment as per local standard of care Participation in a clinical study of another investigational product within 12 weeks prior to randomisation or planning to begin participation during this study Prior participation in the study during a previous pneumonia diagnosis
**Treatment failure**	
Any time on or before day 4	Severe respiratory distress Hypoxaemia WHO IMCI danger signs Missing >2 study drug doses due to vomiting Change in antibiotics prescribed by a study clinician Hospitalisation due to pneumonia (if not initially admitted) Prolonged hospitalisation or re-admission due to pneumonia (if initially admitted) Death
On day 4 only	Axillary temperature >38°C in the absence of a diagnosed co-infection with fever symptoms (eg, malaria)
**Clinical relapse**	
Any time after day 4	Recurrence of signs of pneumonia Signs of severe disease
**NIH DAIDS severity grading**	
Grade 1	Mild event
Grade 2	Moderate event
Grade 3	Severe event
Grade 4	Potentially life-threatening event
Grade 5	Death

DAIDS, Division of AIDS; IMCI, Integrated Management of Childhood Illness; MUAC, mid-upper arm circumference; NIH, National Institutes of Health.

On administration of the initial study drug dose on day 1, all children were observed by study staff for at least 2–8 hours before considering discharge. Children with no fever and a respiratory rate below the enrolment respiratory rate threshold were discharged after 2 hours of observation. Children aged less than 6 months, with moderate malnutrition, or febrile with a negative malaria rapid diagnostic test were monitored inpatient overnight and assessed by study staff for discharge on the morning of day 2. At any time between 2 hours post-enrolment and the morning of day 2, if a child’s condition deteriorated, s/he was hospitalised, the study drug was discontinued and treatment was provided as per local standard of care. If a child showed signs of deterioration resulting in a SAE, a detailed SAE report was completed. The United States National Institutes of Health (NIH) Division of AIDS (DAIDS) adverse event (AE) grading system was used to assess severity of the event (table 1).^8^ All enrolled children requiring hospitalisation for their pneumonia any time between days 1 and 14 were initially treated with benzyl penicillin and gentamycin intravenously as per standard of care at KCH. The study drug was discontinued for any enrolled children who were hospitalised between days 1 and 4. Chest radiographs were obtained at the discretion of study clinicians, and the local principal investigator made the final clinical interpretation. A data safety monitoring board was established to routinely and independently assess child safety throughout the trial. Participants had regular follow-up visits at the study clinic, and if a child missed a visit, the clinical team conducted a home visit to assess the health status of the child.

Medical history, physical exam information, laboratory results and chest radiographs collected at screening, enrolment and during hospitalisation were evaluated. We reviewed all SAE reports for any additional information regarding the child’s hospitalisation, course of treatment and outcome. Reported possible side effects of amoxicillin (ie, fever, gastroenteritis, skin conditions and candidiasis) were also reviewed.

### Patient and public involvement

The University of North Carolina Project Lilongwe community advisory board assisted with community outreach during study recruitment. However, neither the study participants nor the community advisory board were involved in the development of the research question, study design or outcome measures.

## Results

**Figure 1:**
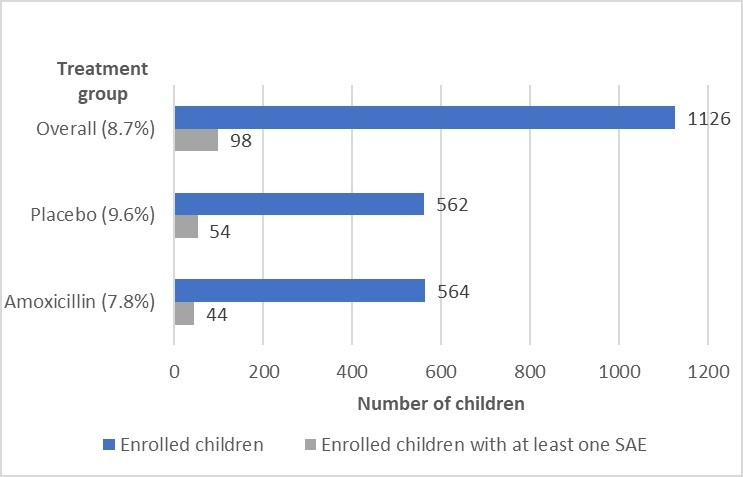
Serious adverse events.

A total of 1126 HIV-uninfected children with fast breathing pneumonia were enrolled with 102 SAEs reported. Among those enrolled, 98 (8.7%) children experienced at least 1 SAE (figure 1), and 4 children had more than 1 SAE. SAEs were evenly distributed between males and females (table 2). Children aged 2–11, 12–23 and 24–59 months accounted for 39.7% (n=39), 35.7% (n=35) and 24.5% (n=24) of all SAEs, respectively. Baseline clinical characteristics at enrolment for those with at least 1 SAE included runny nose (n=47), fever (n=25), diarrhoea by caregiver report (n=9) and malaria (n=5). Only three children were moderately malnourished by MUAC. Of the 98 children with at least 1 SAE, 53 (54.0%) who should have received all three pneumococcal conjugate vaccine doses based on age did receive all three doses and 54 (55.1%) who should have received all three pentavalent vaccine doses based on age did receive all three doses (table 3). Fifty-five (4.9%) SAEs occurred during days 1–4 while receiving study drug, and the remaining 47 (4.2%) SAEs occurred after day 4.

**Table 2:** Baseline clinical presentation at enrolment

	Amoxicillin				Placebo			
	Age 2–6 months	Age 7–11 months	Age 12–23 months	Age 24–59 months	Age 2–6 months	Age 7–11 months	Age 12–23 months	Age 24–59 months
No of children	9	7	18	10	14	9	17	14
Male gender, n (%)	6 (66.7)	4 (57.1)	10 (55.6)	7 (70.0)	6 (42.9)	4 (44.4)	6 (35.3)	6 (42.9)
Malaria present, n (%)	0 (0.0)	1 (14.3)	1 (5.6)	2 (20.0)	0 (0.0)	0 (0.0)	0 (0.0)	1 (7.1)
Diarrhoea (by caretaker assessment), n (%)	1 (11.1)	1 (14.3)	1 (5.6)	0 (0.0)	0 (0.0)	2 (22.2)	2 (11.8)	2 (14.3)
Respiratory rate (breaths/min) 40–49, n (%)	0 (0.0)	0 (0.0)	8 (44.4)	7 (70.0)	0 (0.0)	0 (0.0)	10 (58.8)	10 (71.4)
Respiratory rate (breaths/min) >49, n (%)	9 (100.0)	7 (100.0)	10 (55.6)	3 (30.0)	14 (100.0)	9 (100.0)	7 (41.2)	4 (28.6)
Oxygen saturation 90%–93%, n (%)	0 (0.0)	0 (0.0)	1 (5.6)	0 (0.0)	0 (0.0)	0 (0.0)	0 (0.0)	0 (0.0)
Oxygen saturation >93%, n (%)	9 (100.0)	7 (100.0)	17 (94.4)	10 (100.0)	14 (100.0)	9 (100.0)	17 (100.0)	14 (100.0)
Axillary temperature >38°C, n (%)	1 (11.1)	1 (14.3)	5 (27.8)	4 (40.0)	1 (7.1)	1 (11.1)	3 (17.6)	9 (64.3)
Axillary temperature <38°C, n (%)	8 (88.9)	6 (85.7)	13 (72.2)	6 (60.0)	13 (92.9)	8 (88.9)	14 (82.4)	5 (35.7)
Heart rate (beats/min) Median	156	149	150	136	147	150	145	153
Heart rate (beats/min) Min, Max	129, 170	117, 162	126, 192	130, 156	129, 168	120, 162	133, 163	125, 170
Height/weight Z-score, n	9	7	18	10	14	9	17	14
Height/weight Z-score > −2, n (%)	9 (100.0)	7 (100.0)	17 (94.4)	10 (100.0)	13 (92.9)	9 (100.0)	17 (100.0)	13 (92.9)
Height/weight Z-score −2 and −3, n (%)	0 (0.0)	0 (0.0)	1 (5.6)	0 (0.0)	1 (7.1)	0 (0.0)	0 (0.0)	1 (7.1)
Height/weight Z-score Mean (SD)	1.5 (1.2)	0.0 (1.4)	0.9 (1.5)	0.0 (1.3)	0.9 (1.9)	0.4 (1.0)	0.8 (0.6)	−0.2 (1.1)
Mid-upper arm circumference, n	9	7	18	10	14	9	17	14
Mid-upper arm circumference >125 mm, n (%)	8 (88.9)	7 (100.0)	18 (100.0)	10 (100.0)	12 (85.7)	9 (100.0)	17 (100.0)	14 (100.0)
Mid-upper arm circumference 115–125 mm, n (%)	1 (11.1)	0 (0.0)	0 (0.0)	0 (0.0)	2 (14.3)	0 (0.0)	0 (0.0)	0 (0.0)
Mid-upper arm circumference Median (IQR)	140.6 (8.9)	144.6 (6.4)	153.4 (11.5)	156.2 (14.0)	139.3 (10.6)	146.2 (9.5)	150.4 (9.6)	151.4 (6.9)
Evidence of discharge from ear, n (%)	0 (0.0)	0 (0.0)	0 (0.0)	0 (0.0)	0 (0.0)	0 (0.0)	0 (0.0)	0 (0.0)
Runny nose, n (%)	6 (66.7)	6 (85.7)	8 (44.4)	5 (50.0)	6 (42.9)	4 (44.4)	6 (35.3)	6 (42.9)
Oral thrush, n (%)	0 (0.0)	0 (0.0)	0 (0.0)	0 (0.0)	0 (0.0)	0 (0.0)	0 (0.0)	0 (0.0)
Tender, enlarged lymph node(s) on neck, n (%)	0 (0.0)	0 (0.0)	0 (0.0)	0 (0.0)	0 (0.0)	0 (0.0)	0 (0.0)	0 (0.0)
Stiff neck, n (%)	0 (0.0)	0 (0.0)	0 (0.0)	0 (0.0)	0 (0.0)	0 (0.0)	0 (0.0)	0 (0.0)

**Table 3:** Pneumococcal conjugate and pentavalent vaccines history

	Amoxicillin		Placebo	
	2–3 months*	4–59 months*	2–3 months*	4–59 months*
No of children	4	40	6	48
Pneumococcal conjugate vaccine				
Received three doses, n (%)	1 (25.0)	24 (60.0)	0 (0.0)	29 (60.4)
Received two doses, n (%)	0 (0.0)	3 (7.5)	1 (16.7)	0 (0.0)
Received one dose, n (%)	0 (0.0)	0 (0.0)	0 (0.0)	0 (0.0)
Received 0 doses, n (%)	0 (0.0)	0 (0.0)	0 (0.0)	0 (0.0)
All doses unknown, n (%)	0 (0.0)	13 (32.5)	0 (0.0)	19 (39.6)
Some doses unknown, n (%)				
Received two doses	2 (50.0)	0 (0.0)	5 (83.3)	0 (0.0)
Received one dose	1 (25.0)	0 (0.0)	0 (0.0)	0 (0.0)
Received 0 doses	0 (0.0)	0 (0.0)	0 (0.0)	0 (0.0)
Pentavalent vaccine				
Received three doses, n (%)	1 (25.0)	25 (62.5)	0 (0.0)	29 (60.4)
Received two doses, n (%)	0 (0.0)	2 (5.0)	1 (16.7)	0 (0.0)
Received one dose, n (%)	0 (0.0)	0 (0.0)	0 (0.0)	0 (0.0)
Received 0 doses, n (%)	0 (0.0)	0 (0.0)	0 (0.0)	0 (0.0)
All doses unknown, n (%)	0 (0.0)	13 (32.5)	0 (0.0)	19 (39.6)
Some doses unknown, n (%)				
Received two doses	2 (50.0)	0 (0.0)	5 (83.3)	0 (0.0)
Received one dose	1 (25.0)	0 (0.0)	0 (0.0)	0 (0.0)
Received 0 doses	0 (0.0)	0 (0.0)	0 (0.0)	0 (0.0)

*Age groups reflect recommended vaccine dosing schedules:<3 months should have received one dose;>3 months should have received all three doses.

**Figure 2:**
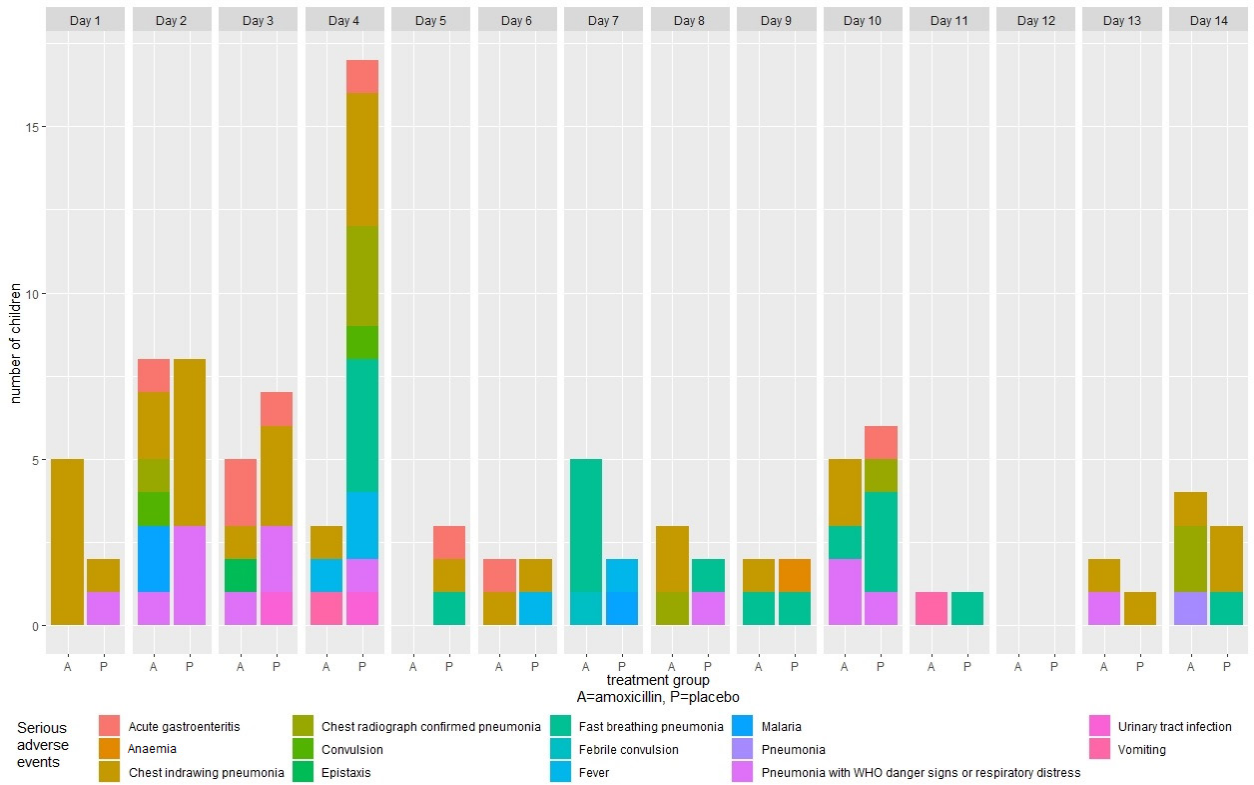
Serious adverse events by visit day and treatment group.

Of the 102 SAEs, 77 (75.5%; p=<0.01) were pneumonia-related SAEs (table 4). Of the 77 pneumonia-related SAEs, 39 (27 in the placebo group and 12 in the amoxicillin group) occurred during days 1–4 while receiving study drug, and 38 (16 in the placebo group and 22 in the amoxicillin group) occurred after day 4 (figure 2). Types of pneumonia-related SAEs included progression to chest indrawing pneumonia (n=35; 45.5%), persistence of fast breathing pneumonia (n=19; 24.7%), progression to pneumonia with respiratory distress or WHO general danger signs (n=14; 18.2%), chest radiograph confirmed pneumonia (n=8; 10.4%) and one pneumonia case with no additional data regarding type of pneumonia. Of the 77 pneumonia-related SAEs, 61 (79.2%) cases occurred in children aged <2 years (table 5). Fifty (64.9%) of the pneumonia-related SAEs were NIH DAIDs severity grade 3 (severe) and 27 (35.0%) were severity grade 2 (moderate) (S1 Appendix, supporting material). There were no grade 4 (life-threatening) SAEs. All children recovered without sequelae. There were no deaths.

10.1136/bmjresp-2019-000415.supp1 Supplementary data

**Table 4:** Serious adverse events by treatment group

	Amoxicillin	Placebo	Overall
Serious adverse events per group	46	56	102
Serious adverse events (can be multiple events per child)			
Pneumonia-related, n (%)*	34 (73.9)	43 (76.8)	77 (75.5)
Chest indrawing pneumonia	17 (50.0)	18 (41.9)	35 (45.5)
Fast breathing pneumonia	7 (20.6)	12 (27.9)	19 (24.7)
Pneumonia with respiratory distress or WHO general danger signs	5 (14.7)	9 (20.9)	14 (18.1)
Chest radiograph confirmed pneumonia†	4 (11.8)	4 (9.3)	8 (10.4)
Pneumonia‡	1 (3.0)	0 (0.0)	1 (1.3)
Non-pneumonia related, n (%)*	12 (26.0)	13 (23.2)	25 (24.5)
Acute gastroenteritis	4 (33.3)	4 (30.8)	8 (32.0)
Fever	1 (8.3)	4 (30.8)	5 (20.0)
Malaria	2 (16.7)	1 (7.7)	3 (12.0)
Convulsion	1 (8.3)	1 (7.7)	2 (8.0)
Urinary tract infection	0 (0.0)	2 (15.4)	2 (8.0)
Vomiting	2 (16.7)	0 (0.0)	2 (8.0)
Anaemia	0 (0.0)	1 (7.7)	1 (4.0)
Epistaxis	1 (8.3)	0 (0.0)	1 (4.0)
Febrile convulsion	1 (8.3)	0 (0.0)	1 (4.0)

*Percentages for ‘Amoxicillin’ and ‘Placebo’ columns reflect total number of children within each category by treatment group. Percentages for ‘Overall’ column reflect total number of serious adverse event cases.

†Chest radiograph performed due to persistent fever leading to identification of pneumonia.

‡Event reported from an outside hospital and hospital records did not specify type of pneumonia.

**Table 5:** Serious adverse event by age and treatment group

Age group (months)	Amoxicillin				Placebo			
	2–6	7–11	12–23	24–59	2–6	7–11	12–23	24–59
Serious adverse events, n	9	7	19	11	14	10	17	15
Fast breathing pneumonia, n (%)	0 (0.0%)	1 (0.2%)	4 (0.7%)	2 (0.4%)	3 (0.5%)	1 (0.2%)	5 (0.9%)	3 (0.5%)
Chest indrawing pneumonia, n (%)	6 (1.1%)	1 (0.2%)	7 (1.2%)	3 (0.5%)	10 (1.8%)	1 (0.2%)	6 (1.1%)	1 (0.2%)
Pneumonia with respiratory distress or general danger signs, n (%)	0 (0.0%)	1 (0.2%)	3 (0.5%)	1 (0.2%)	1 (0.2%)	3 (0.5%)	3 (0.5%)	2 (0.4%)
Chest radiograph confirmed pneumonia, n (%)*	1 (0.2%)	1 (0.2%)	2 (0.4%)	0 (0.0%)	0 (0.0%)	0 (0.0%)	0 (0.0%)	4 (0.7%)
Pneumonia, n (%)	1 (0.2%)	0 (0.0%)	0 (0.0%)	0 (0.0%)	0 (0.0%)	0 (0.0%)	0 (0.0%)	0 (0.0%)
Acute gastroenteritis, n (%)	1 (0.2%)	1 (0.2%)	2 (0.4%)	0 (0.0%)	0 (0.0%)	3 (0.5%)	1 (0.2%)	0 (0.0%)
Fever, n (%)	0 (0.0%)	1 (0.2%)	0 (0.0%)	0 (0.0%)	0 (0.0%)	2 (0.4%)	0 (0.0%)	2 (0.4%)
Malaria, n (%)	0 (0.0%)	1 (0.2%)	0 (0.0%)	1 (0.2%)	0 (0.0%)	0 (0.0%)	1 (0.2%)	0 (0.0%)
Convulsion, n (%)	0 (0.0%)	0 (0.0%)	0 (0.0%)	1 (0.2%)	0 (0.0%)	0 (0.0%)	1 (0.2%)	0 (0.0%)
Urinary tract infection, n (%)	0 (0.0%)	0 (0.0%)	0 (0.0%)	0 (0.0%)	0 (0.0%)	0 (0.0%)	0 (0.0%)	2 (0.4%)
Vomiting, n (%)	0 (0.0%)	0 (0.0%)	1 (0.2%)	1 (0.2%)	0 (0.0%)	0 (0.0%)	0 (0.0%)	0 (0.0%)
Anaemia, n (%)	0 (0.0%)	0 (0.0%)	0 (0.0%)	0 (0.0%)	0 (0.0%)	0 (0.0%)	0 (0.0%)	1 (0.2%)
Epistaxis, n (%)	0 (0.0%)	0 (0.0%)	0 (0.0%)	1 (0.2%)	0 (0.0%)	0 (0.0%)	0 (0.0%)	0 (0.0%)
Febrile convulsion, n (%)	0 (0.0%)	0 (0.0%)	0 (0.0%)	1 (0.2%)	0 (0.0%)	0 (0.0%)	0 (0.0%)	0 (0.0%)

*Chest radiograph performed due to persistent fever leading to identification of pneumonia.

There were 25 non-pneumonia-related SAEs: acute gastroenteritis (n=8); fever (n=5); malaria (n=3); convulsion (n=3); and other (n=6; urinary tract infections (n=2), vomiting (n=2); epistaxis (n=1); anaemia (n=1)). Of the 25 non-pneumonia-related SAEs, 16 occurred between days 1 and 4 (7 in the placebo group and 9 in the amoxicillin group), and 9 occurred after day 4 (6 in the placebo group and 3 in the amoxicillin group). The NIH DAIDs severity grade for 15 of the non-pneumonia-related SAEs was grade 3; 8 were grade 2 SAEs. All recovered without sequelae. There were no deaths. The average duration of hospitalisation for both pneumonia-related and non-pneumonia-related SAEs was 10 days, and was similar in both the placebo and amoxicillin groups (S2 Appendix, supporting material).

When reviewing the relationship of SAE to study drug, 4 gastroenteritis cases and 1 fever were noted as “possibly related” in the amoxicillin group (10.9%). All other SAEs were assessed as not related to study drug (S3 Appendix, supporting material). A total of 200 AEs in the amoxicillin group (n=564) were reviewed and 77 (13.7%) AEs were identified as possibly amoxicillin-related side effects, of which 52 (67.5%) were gastroenteritis. These cases did not require hospitalisation and were not reported as SAEs. Rash was reported in 8 (10.4%) children, and candidiasis in 4 (5.2%) children in the amoxicillin group (S3 Appendix, supporting material). For comparison, in the placebo group (n=562), there were 241 (42.9%) AEs, 47 (19.5%) of which were gastroenteritis, 12 (5.0%) rash and 1 (0.4%) candidiasis. In contrast to children with SAEs, the 1028 children (520 in the amoxicillin group, 508 in the placebo group) without SAEs were predominantly greater than 1 year of age (65.7%, 675/1028) and had slightly higher rates of malaria (13.2%, 136/1028) (S4 Appendix, supporting material). There were no significant differences in all other baseline characteristics between children with SAEs compared with those without.

## Discussion

In this fast breathing pneumonia clinical trial, SAEs were infrequent. In addition, the number of children who required hospitalisation due to a SAE while receiving study drug or placebo was low, even among children aged <2 years. None of the SAEs were life-threatening or resulted in death. The low rate of SAEs in our trial may be attributed to the high level of supportive care and close monitoring of children during the study, particularly between days 1 and 4. It could also be argued that the low rate of SAEs may be a consequence of the trial’s strict study eligibility criteria that included relatively low-risk children and excluded HIV-seropositive children as well as any child exhibiting signs of severe disease, anaemia or acute malnourishment. Another fast breathing pneumonia clinical trial, also comparing 3 days of amoxicillin to placebo in children, recently completed in Karachi, Pakistan and a similar secondary analysis of their SAEs may provide additional insight into the generalisability of our results.

A key aim in reviewing SAEs within this trial was to assess potential adverse impacts of amoxicillin use for treatment of childhood pneumonia, particularly since the diagnosis of pneumonia was based solely on clinical signs and did not involve any laboratory or radiographic confirmation. Common harms from antibiotics are poorly quantified and often are not well reported in clinical trials.^6^ Within our trial there were 5 SAEs, 4 cases of gastroenteritis and 1 fever, determined to be possibly related to receiving amoxicillin. Non-pneumonia-related SAEs were almost evenly distributed between the placebo and amoxicillin groups (placebo 13/25 vs amoxicillin 12/25), with the most common non-pneumonia-related SAE being acute gastroenteritis. The low number of SAEs in the amoxicillin group supports the general safety of amoxicillin use in children.^7^ In a meta-analysis reviewing randomised trials of amoxicillin and/or amoxicillin–clavulanic acid, diarrhoea and candidiasis were identified as the most common associated harms.^6^ Our secondary analysis is limited because it focuses primarily on SAEs. We did review AEs; however, we did not look at all AEs, but rather only those that could be possible side effects of amoxicillin. Gastroenteritis was the most commonly reported side effect in the amoxicillin group but attribution to amoxicillin was not examined. A more comprehensive review of all AEs may provide additional information confirming which diarrhoea and vomiting events might be attributed to amoxicillin use, as well as other reported possible side effects, during the trial.

The number of children with 2 or more SAEs was low; only 4 out of 98 children with at least 1 SAE had multiple SAEs. Although most initial SAE hospitalisations were due to progression or recurrent pneumonia, only 4% of children were readmitted for additional treatment of pneumonia or other severe disease. In low resource settings, the months following hospital discharge carry significant risk for morbidity and mortality, with pneumonia identified as one of the baseline variables associated with post-discharge mortality.^9^ This highlights a major limitation in this analysis. Follow-up of children post-discharge was restricted to the study participation period of 2 weeks, with a 2-day visit window if the child missed day 14. We do not have additional outcome data after day 14 and are therefore unable to assess if any children were readmitted or died within 30 days of enrolment at KCH or other local healthcare facilities. We also are not able to assess any long-term potential adverse impacts of amoxicillin use (eg, microbiome, growth, antimicrobial resistance). Additional limitations of this secondary analysis include our small sample size and the strict eligibility criteria of the trial. Our results may not be generalisable across other settings or non-trial conditions and should be interpreted within that context. The children enrolled in this trial were non-severe pneumonia cases without HIV and very few comorbidities or complications. This may have ultimately biased the number of SAEs.

## Conclusion

The overall incidence of SAEs among children in this fast breathing pneumonia clinical trial was low. This also held true among the children receiving placebo for their fast breathing pneumonia. Only 5 SAEs were possibly related to receipt of amoxicillin. Enrolled children aged 2–23 months represented the greatest proportion of those with any SAE or a pneumonia-related SAE. Our analysis and findings help to underscore the importance of adequate and complete safety monitoring and reporting in phase 4 clinical trials, particularly those involving children. The results of this analysis demonstrate that it is possible to safely conduct a clinical trial of this magnitude with placebo in a paediatric group. While this could be credited in part to the trial eligibility criteria and close monitoring by the clinical research site, it should be noted that there were no life-threatening pneumonia SAEs or deaths, even among those in the placebo group. The dissemination of this data is useful to better understand the balance of risks versus benefits of antibiotic treatment in young children, even with a well-established and commonly used antibiotic such as amoxicillin.
