# Supplementary material for: Analysis of serious adverse events in a paediatric fast breathing pneumonia clinical trial in Malawi
Source: BMJ Open Respir Res. 2019 Sep 3;6(1):e000415. doi: 10.1136/bmjresp-2019-000415 (PMC6733335; doi:10.1136/bmjresp-2019-000415)
Supplement: Supplementary data [file bmjresp-2019-000415supp001.pdf]

Supporting materials appendices

S1 Appendix. Serious adverse event severity by treatment group

	Amoxicillin	Placebo	Overall
n SAEs	46	56	102
Severity			
Pneumonia-related, n	34	43	77
Grade 1 - Mild	0	0	0
Grade 2 - Moderate	12	15	27
Grade 3 - Severe	22	28	50
Grade 4 - Life-threatening	0	0	0
Non-pneumonia related, n	12	13	25
Grade 1 - Mild	1	0	1
Grade 2 - Moderate	3	5	8
Grade 3 - Severe	8	7	15
Grade 4 - Life-threatening	0	1	1

SAE=serious adverse event

S2 Appendix. Length of hospitalization by age and treatment group

	Amoxicillin								
	n SAEs	Mean (sd)	1 Day	2 Days	3 Days	4 Days	5 Days	6 Days	7+ days
**Age 2-6 months**									
Fast breathing pneumonia	0	--	--	--	--	--	--	--	--
Chest indrawing pneumonia	6	10.3 (4.0)	0 (0.0%)	0 (0.0%)	0 (0.0%)	0 (0.0%)	1 (16.7%)	0 (0.0%)	5 (83.3%)
Pneumonia with WHO general danger sign or respiratory distress	0	--	--	--	--	--	--	--	--
Chest radiograph confirmed pneumonia	1	13.0 (--)	0 (0.0%)	0 (0.0%)	0 (0.0%)	0 (0.0%)	0 (0.0%)	0 (0.0%)	1 (100.0%)

Pneumonia	1	1.0 (--)	1 (100.0%)	0 (0.0%)	0 (0.0%)	0 (0.0%)	0 (0.0%)	0 (0.0%)	0 (0.0%)
Acute gastroenteritis	1	12.0 (--)	0 (0.0%)	0 (0.0%)	0 (0.0%)	0 (0.0%)	0 (0.0%)	0 (0.0%)	1 (100.0%)
Fever	0	--	--	--	--	--	--	--	--
Malaria	0	--	--	--	--	--	--	--	--
Convulsion	0	--	--	--	--	--	--	--	--
Urinary tract infection	0	--	--	--	--	--	--	--	--
Vomiting	0	--	--	--	--	--	--	--	--
Anemia	0	--	--	--	--	--	--	--	--
Epistaxis	0	--	--	--	--	--	--	--	--
Febrile convulsion	0	--	--	--	--	--	--	--	--
**Age 7-11 months**									
Fast breathing pneumonia	1	34.0 (--)	0 (0.0%)	0 (0.0%)	0 (0.0%)	0 (0.0%)	0 (0.0%)	0 (0.0%)	1 (100.0%)
Chest indrawing pneumonia	1	10.0 (--)	0 (0.0%)	0 (0.0%)	0 (0.0%)	0 (0.0%)	0 (0.0%)	0 (0.0%)	1 (100.0%)
Pneumonia with WHO general danger sign or respiratory distress	1	12.0 (--)	0 (0.0%)	0 (0.0%)	0 (0.0%)	0 (0.0%)	0 (0.0%)	0 (0.0%)	1 (100.0%)
Chest radiograph confirmed pneumonia	1	7.0 (--)	0 (0.0%)	0 (0.0%)	0 (0.0%)	0 (0.0%)	0 (0.0%)	0 (0.0%)	1 (100.0%)
Pneumonia	0	--	--	--	--	--	--	--	--
Acute gastroenteritis	1	9.0 (--)	0 (0.0%)	0 (0.0%)	0 (0.0%)	0 (0.0%)	0 (0.0%)	0 (0.0%)	1 (100.0%)
Fever	1	11.0 (--)	0 (0.0%)	0 (0.0%)	0 (0.0%)	0 (0.0%)	0 (0.0%)	0 (0.0%)	1 (100.0%)
Malaria	1	13.0 (--)	0 (0.0%)	0 (0.0%)	0 (0.0%)	0 (0.0%)	0 (0.0%)	0 (0.0%)	1 (100.0%)
Convulsion	0	--	--	--	--	--	--	--	--
Urinary tract infection	0	--	--	--	--	--	--	--	--
Vomiting	0	--	--	--	--	--	--	--	--
Anemia	0	--	--	--	--	--	--	--	--
Epistaxis	0	--	--	--	--	--	--	--	--
Febrile convulsion	0	--	--	--	--	--	--	--	--
**Age 12-23 months**									
Fast breathing pneumonia	4	7.3 (2.1)	0 (0.0%)	0 (0.0%)	0 (0.0%)	0 (0.0%)	1 (25.0%)	1 (25.0%)	2 (50.0%)
Chest indrawing pneumonia	7	12.1 (2.9)	0 (0.0%)	0 (0.0%)	0 (0.0%)	0 (0.0%)	0 (0.0%)	0 (0.0%)	7 (100.0%)
Pneumonia with WHO general danger sign or respiratory distress	3	5.3 (1.5)	0 (0.0%)	0 (0.0%)	0 (0.0%)	1 (33.3%)	1 (33.3%)	0 (0.0%)	1 (33.3%)

Chest radiograph confirmed pneumonia	2	9.0 (1.4)	0 (0.0%)	0 (0.0%)	0 (0.0%)	0 (0.0%)	0 (0.0%)	0 (0.0%)	2 (100.0%)
Pneumonia	0	--	--	--	--	--	--	--	--
Acute gastroenteritis	2	9.0 (5.7)	0 (0.0%)	0 (0.0%)	0 (0.0%)	0 (0.0%)	1 (50.0%)	0 (0.0%)	1 (50.0%)
Fever	0	--	--	--	--	--	--	--	--
Malaria	0	--	--	--	--	--	--	--	--
Convulsion	0	--	--	--	--	--	--	--	--
Urinary tract infection	0	--	--	--	--	--	--	--	--
Vomiting	1	22.0 (--)	0 (0.0%)	0 (0.0%)	0 (0.0%)	0 (0.0%)	0 (0.0%)	0 (0.0%)	1 (100.0%)
Anemia	0	--	--	--	--	--	--	--	--
Epistaxis	0	--	--	--	--	--	--	--	--
Febrile convulsion	0	--	--	--	--	--	--	--	--
**Age 24-59 months**									
Fast breathing pneumonia	2	8.0 (0.0)	0 (0.0%)	0 (0.0%)	0 (0.0%)	0 (0.0%)	0 (0.0%)	0 (0.0%)	2 (100.0%)
Chest indrawing pneumonia	3	4.7 (2.1)	0 (0.0%)	0 (0.0%)	1 (33.3%)	1 (33.3%)	0 (0.0%)	0 (0.0%)	1 (33.3%)
Pneumonia with WHO general danger sign or respiratory distress	1	27.0 (--)	0 (0.0%)	0 (0.0%)	0 (0.0%)	0 (0.0%)	0 (0.0%)	0 (0.0%)	1 (100.0%)
Chest radiograph confirmed pneumonia	0	--	--	--	--	--	--	--	--
Pneumonia	0	--	--	--	--	--	--	--	--
Acute gastroenteritis	0	--	--	--	--	--	--	--	--
Fever	0	--	--	--	--	--	--	--	--
Malaria	1	13.0 (--)	0 (0.0%)	0 (0.0%)	0 (0.0%)	0 (0.0%)	0 (0.0%)	0 (0.0%)	1 (100.0%)
Convulsion	1	378.0 (--)	0 (0.0%)	0 (0.0%)	0 (0.0%)	0 (0.0%)	0 (0.0%)	0 (0.0%)	1 (100.0%)
Urinary tract infection	0	--	--	--	--	--	--	--	--
Vomiting	1	11.0 (--)	0 (0.0%)	0 (0.0%)	0 (0.0%)	0 (0.0%)	0 (0.0%)	0 (0.0%)	1 (100.0%)
Anemia	0	--	--	--	--	--	--	--	--
Epistaxis	1	3.0 (--)	0 (0.0%)	0 (0.0%)	1 (100.0%)	0 (0.0%)	0 (0.0%)	0 (0.0%)	0 (0.0%)
Febrile convulsion	1	10.0 (--)	0 (0.0%)	0 (0.0%)	0 (0.0%)	0 (0.0%)	0 (0.0%)	0 (0.0%)	1 (100.0%)
	**Placebo**								

	n SAEs	Mean (sd)	1 Day	2 Days	3 Days	4 Days	5 Days	6 Days	7+ days
**Age 2-6 months**									
Fast breathing pneumonia	3	7.0 (2.6)	0 (0.0%)	0 (0.0%)	0 (0.0%)	0 (0.0%)	1 (33.3%)	1 (33.3%)	1 (33.3%)
Chest indrawing pneumonia	10	11.7 (1.1)	0 (0.0%)	0 (0.0%)	0 (0.0%)	0 (0.0%)	0 (0.0%)	0 (0.0%)	10 (100.0%)
Pneumonia with WHO general danger sign or respiratory distress	1	13.0 (--)	0 (0.0%)	0 (0.0%)	0 (0.0%)	0 (0.0%)	0 (0.0%)	0 (0.0%)	1 (100.0%)
Chest radiograph confirmed pneumonia	0	--	--	--	--	--	--	--	--
Pneumonia	0	--	--	--	--	--	--	--	--
Acute gastroenteritis	0	--	--	--	--	--	--	--	--
Fever	0	--	--	--	--	--	--	--	--
Malaria	0	--	--	--	--	--	--	--	--
Convulsion	0	--	--	--	--	--	--	--	--
Urinary tract infection	0	--	--	--	--	--	--	--	--
Vomiting	0	--	--	--	--	--	--	--	--
Anemia	0	--	--	--	--	--	--	--	--
Epistaxis	0	--	--	--	--	--	--	--	--
Febrile convulsion	0	--	--	--	--	--	--	--	--
**Age 7-11 months**									
Fast breathing pneumonia	1	10.0 (--)	0 (0.0%)	0 (0.0%)	0 (0.0%)	0 (0.0%)	0 (0.0%)	0 (0.0%)	1 (100.0%)
Chest indrawing pneumonia	1	3.0 (--)	0 (0.0%)	0 (0.0%)	1 (100.0%)	0 (0.0%)	0 (0.0%)	0 (0.0%)	0 (0.0%)
Pneumonia with WHO general danger sign or respiratory distress	3	10.7 (2.5)	0 (0.0%)	0 (0.0%)	0 (0.0%)	0 (0.0%)	0 (0.0%)	0 (0.0%)	3 (100.0%)
Chest radiograph confirmed pneumonia	0	--	--	--	--	--	--	--	--
Pneumonia	0	--	--	--	--	--	--	--	--
Acute gastroenteritis	3	7.3 (4.0)	0 (0.0%)	0 (0.0%)	0 (0.0%)	0 (0.0%)	2 (66.7%)	0 (0.0%)	1 (33.3%)
Fever	2	10.0 (2.8)	0 (0.0%)	0 (0.0%)	0 (0.0%)	0 (0.0%)	0 (0.0%)	0 (0.0%)	2 (100.0%)
Malaria	0	--	--	--	--	--	--	--	--
Convulsion	0	--	--	--	--	--	--	--	--

Urinary tract infection	0	--	--	--	--	--	--	--	--
Vomiting	0	--	--	--	--	--	--	--	--
Anemia	0	--	--	--	--	--	--	--	--
Epistaxis	0	--	--	--	--	--	--	--	--
Febrile convulsion	0	--	--	--	--	--	--	--	--
**Age 12-23 months**									
Fast breathing pneumonia	5	7.4 (2.2)	0 (0.0%)	0 (0.0%)	0 (0.0%)	0 (0.0%)	1 (20.0%)	0 (0.0%)	4 (80.0%)
Chest indrawing pneumonia	6	10.3 (3.4)	0 (0.0%)	0 (0.0%)	0 (0.0%)	1 (16.7%)	0 (0.0%)	0 (0.0%)	5 (83.3%)
Pneumonia with WHO general danger sign or respiratory distress	3	12.0 (1.0)	0 (0.0%)	0 (0.0%)	0 (0.0%)	0 (0.0%)	0 (0.0%)	0 (0.0%)	3 (100.0%)
Chest radiograph confirmed pneumonia	0	--	--	--	--	--	--	--	--
Pneumonia	0	--	--	--	--	--	--	--	--
Acute gastroenteritis	1	10.0 (--)	0 (0.0%)	0 (0.0%)	0 (0.0%)	0 (0.0%)	0 (0.0%)	0 (0.0%)	1 (100.0%)
Fever	0	--	--	--	--	--	--	--	--
Malaria	1	8.0 (--)	0 (0.0%)	0 (0.0%)	0 (0.0%)	0 (0.0%)	0 (0.0%)	0 (0.0%)	1 (100.0%)
Convulsion	1	13.0 (--)	0 (0.0%)	0 (0.0%)	0 (0.0%)	0 (0.0%)	0 (0.0%)	0 (0.0%)	1 (100.0%)
Urinary tract infection	0	--	--	--	--	--	--	--	--
Vomiting	0	--	--	--	--	--	--	--	--
Anemia	0	--	--	--	--	--	--	--	--
Epistaxis	0	--	--	--	--	--	--	--	--
Febrile convulsion	0	--	--	--	--	--	--	--	--
**Age 24-59 months**									
Fast breathing pneumonia	3	9.7 (2.3)	0 (0.0%)	0 (0.0%)	0 (0.0%)	0 (0.0%)	0 (0.0%)	0 (0.0%)	3 (100.0%)
Chest indrawing pneumonia	1	14.0 (--)	0 (0.0%)	0 (0.0%)	0 (0.0%)	0 (0.0%)	0 (0.0%)	0 (0.0%)	1 (100.0%)
Pneumonia with WHO general danger sign or respiratory distress	2	4.5 (2.1)	0 (0.0%)	0 (0.0%)	1 (50.0%)	0 (0.0%)	0 (0.0%)	1 (50.0%)	0 (0.0%)
Chest radiograph confirmed pneumonia	4	17.8 (15.0)	0 (0.0%)	0 (0.0%)	0 (0.0%)	0 (0.0%)	0 (0.0%)	0 (0.0%)	4 (100.0%)
Pneumonia	0	--	--	--	--	--	--	--	--
Acute gastroenteritis	0	--	--	--	--	--	--	--	--
Fever	2	10.0 (1.4)	0 (0.0%)	0 (0.0%)	0 (0.0%)	0 (0.0%)	0 (0.0%)	0 (0.0%)	2 (100.0%)

Malaria	0	--	--	--	--	--	--	--	--
Convulsion	0	--	--	--	--	--	--	--	--
Urinary tract infection	2	11.5 (0.7)	0 (0.0%)	0 (0.0%)	0 (0.0%)	0 (0.0%)	0 (0.0%)	0 (0.0%)	2 (100.0%)
Vomiting	0	--	--	--	--	--	--	--	--
Anemia	1	11.0 (--)	0 (0.0%)	0 (0.0%)	0 (0.0%)	0 (0.0%)	0 (0.0%)	0 (0.0%)	1 (100.0%)
Epistaxis	0	--	--	--	--	--	--	--	--
Febrile convulsion	0	--	--	--	--	--	--	--	--

SAE=serious adverse event; sd=standard deviation; WHO=World Health Organization

S3 Appendix. Adverse event and serious adverse event relationship to study drug

	Amoxicillin		Placebo		Overall
	N	%	N	%	
Number enrolled, N	564		562		1126
Number of serious adverse events, n[1]	46	8.2	56		102
Not related	42	91.3	52	92.9	94
Possibly related	5	10.9	4	7.1	9
Probably related	0	0.0	0	0.0	0
Probably not related	0	0.0	0	0.0	0
	N	%	N	%	
Number of adverse events	200	45.4	241	54.6	441
Relationship to study drug[1]					
Not related	191	95.5	230	95.4	421
Possibly related	3	1.5	5	2.1	8
Probably	0	0.0	1	0.4	1
Probably not	6	3.0	5	2.1	11
	N	%	N	%	
Number of children with possible amoxicillin-related side-effect adverse events[2]	77	13.7	241	0.4	160
Skin conditions[3]	22	28.6	35	14.5	56

Eczema	0	0.0	6	2.5	6
Ecthyma	1	4.5	0	0.0	1
Folliculitis	0	0.0	1	0.4	1
Furunculosis	0	0.0	1	0.4	1
Impetigo	1	4.5	2	0.8	3
Urticaria	1	4.5	4	1.7	5
Rash	8	10.4	12	5.0	20
Larva migrans	1	4.5	0	0.0	1
Miliaria	2	9.1	2	0.8	4
Ulcers/sores	3	13.6	5	2.1	8
Tenia capitis	3	13.6	2	0.8	5
Tenia corporis	1	4.5	0	0.0	1
Gastroenteritis[3]	52	67.5	47	19.5	99
Acute gastroenteritis	48	92.3	41	17.0	89
Vomiting	2	3.8	2	0.8	4
Diarrhea	2	3.8	4	1.7	5
Candidiasis[3]	4	5.2	1	0.4	5

1. Percentages based on total number of adverse events and serious adverse events in each group.
2. Only applicable to the amoxicillin group: percentages based on total number children with possible amoxicillin-related side effect adverse events.
3. For the amoxicillin group, percentages based on total number of possible amoxicillin-related side effect adverse events. For the placebo group, percentages reflect total number of cases amongst those with an adverse event.

S4a Appendix. Children without a serious adverse event, baseline characteristics

	Amoxicillin N=520	Placebo N=508
Age (months), mean (sd)	21.4 (15.3)	22.0 (15.0)
2 - 6 months, n (%)	103 (19.8%)	96 (18.9%)
7 - 11 months, n (%)	77 (14.8%)	77 (15.2%)
12 - 35 months, n (%)	234 (45.0%)	230 (45.3%)
36 - 59 months, n (%)	106 (20.4%)	105 (20.7%)
>59 months, n (%)	0 (0.0%)	0 (0.0%)
Gender		
Male, n (%)	244 (46.9%)	232 (45.7%)
Female, n (%)	276 (53.1%)	276 (54.3%)
Weight (kg), n	519	508
Mean (sd)	10.5 (2.7)	10.6 (2.6)
Height/length (cm), n	519	508
Mean (sd)	78.4 (12.4)	79.0 (12.1)
Height/weight Z-score, n (%)	519	508
Greater than -2	515 (99.0%)	500 (98.4%)
Between -2 and -3	4 (0.8%)	7 (1.4%)
Less than -3	0 (0.0%)	1 (0.2%)
Mean (sd)	0.5 (1.2)	0.4 (1.2)
Mid-Upper Arm Circumference (mm), n	519	508
>125	491 (94.4%)	470 (92.5%)
115 - 125	28 (5.4%)	38 (7.5%)
<115	0 (0.0%)	0 (0.0%)
Mean (sd)	151.2 (11.3)	151.0 (12.0)

sd=standard deviation; kg=kilograms; cm=centimeters; mm=millimeters

S4b Appendix. Children without a serious adverse event, physical exam and laboratory results

	Amoxicillin N=520	Placebo N=508
Respiratory rate (breaths/min)[1]		
<40, n (%)	0 (0.0%)	0 (0.0%)
40 - 49, n (%)	220 (42.3%)	213 (41.9%)
>49, n (%)	300 (57.7%)	295 (58.1%)
Unknown, n (%)	0 (0.0%)	0 (0.0%)
Oxygen saturation[1]		
< 90%, n (%)	0 (0.0%)	0 (0.0%)
90% - 93%, n (%)	0 (0.0%)	0 (0.0%)
> 93%, n (%)	520 (100.0%)	508 (100.0%)
Unknown, n (%)	0 (0.0%)	0 (0.0%)
Axillary temperature[1]		
≥38 °C, n (%)	167 (32.1%)	143 (28.1%)
<38 °C, n (%)	353 (67.9%)	365 (71.9%)
Unknown, n (%)	0 (0.0%)	0 (0.0%)
Heart rate (beats/min)[1]		
Median	147	147
Min, Max	101, 190	101, 196
HemoCue		
Mean (sd)	10.8 (1.2)	10.9 (1.2)
Malaria		
Positive, n (%)	71 (13.7%)	65 (12.8%)
Negative, n (%)	449 (86.3%)	443 7.2%)

1. Highest value between screening and enrollment

S4c Appendix. Children without a serious adverse event, respiratory rate frequencies by age and treatment group

	Amoxicillin N=520	Placebo N=508
Age 2-11 months		
R^R <50	0 (0.0%)	0 (0.0%)
RR 50-59	1 (0.6%)	0 (0.0%)
RR ≥60	179 (99.4%)	173 (100.0%)
Age 12-59 months		
RR <40	219 (64.4%)	213 (63.6%)
RR 40-49	106 (31.2%)	110 (32.8%)
RR ≥50	15 (4.4%)	12 (3.6%)

RR = respiratory rate
